# *EGFLAM* Pathogenic Variants and Congenital Stationary Night Blindness

**DOI:** 10.1001/jamaophthalmol.2025.4888

**Published:** 2025-12-04

**Authors:** Sanja Boranijasevic, Vasily Smirnov, Julien Navarro, Martha Tjon-Fo-Sang, Christel Condroyer, Lonneke Haer-Wigman, Aline Antonio, Claire-Marie Dhaenens, Virginie J. M. Verhoeven, José-Alain Sahel, L. Ingeborgh van den Born, Sabine Defoort, Isabelle Audo, Christina Zeitz

**Affiliations:** 1 Sorbonne Université, INSERM, CNRS, Institut de la Vision, Paris, France; 2 U1172-LilNCog-Lille Neuroscience & Cognition, Univ. Lille, Inserm, CHU Lille, Lille, France; 3 Exploration de la Vision et Neuro-Ophtalmologie, CHU de Lille, Lille, France; 4 The Rotterdam Eye Hospital and Rotterdam Ophthalmic Institute, Rotterdam, the Netherlands; 5 Department of Human Genetics, Radboud University Medical Centre, Nijmegen, the Netherlands; 6 Research Institute for Medical Innovation, Radboud University Medical Centre, Nijmegen, the Netherlands; 7 Department of Ophthalmology, Erasmus MC, University Medical Centre Rotterdam, Rotterdam, the Netherlands; 8 Department of Clinical Genetics, Erasmus MC, University Medical Centre Rotterdam, Rotterdam, the Netherlands; 9 Centre Hospitalier National d’Ophtalmologie des Quinze-Vingts, Centre de Référence Maladies Rares REFERET and INSERM-DGOS CIC 1423, Paris, France; 10 Department of Ophthalmology, The University of Pittsburgh School of Medicine, Pittsburgh, Pennsylvania; 11 Sorbonne Université, Inserm, CNRS, Institut de la Vision, Paris, France

## Abstract

**Importance:**

Congenital stationary night blindness (CSNB) is a clinically and genetically heterogeneous inherited retinal disorder (IRD), and in many complete CSNB (cCSNB) cases, the underlying genetic cause remains unknown. Uncovering the genetic defects of IRDs helps to refine diagnostic methods and supports the development of specific therapeutic approaches.

**Objective:**

To describe the phenotype and the underlying gene defect in patients with cCSNB from 2 unrelated families.

**Design, Setting and Participants:**

This retrospective case series was conducted from January 2023 to July 2025. Data for 3 patients from cohorts of genetically unsolved IRD cases in France (n = 140 for CSNB) and the Netherlands (n = 2730 for IRD) were analyzed clinically and genetically.

**Exposures:**

Complete ocular examination, including multimodal retinal imaging and full-field electroretinography (ffERG) incorporating the International Society for Clinical Electrophysiology of Vision standards and multimodal retinal imaging, were performed. Gene defects were identified by genome sequencing (GS) and exome sequencing (ES).

**Main Outcomes and Measures:**

The main outcome was a gene defect, *EGFLAM*, underlying cCSNB. Measures included phenotyping, GS, ES, Sanger sequencing, and cosegregation analysis.

**Results:**

The series included 3 patients from 2 unrelated families of Moroccan ancestry showing high myopia, reduced visual acuity, and night blindness. Retinal imaging depicted myopic changes. ffERG revealed electronegative Schubert-Bornschein configuration in keeping with cCSNB with ON-bipolar cell dysfunction. Patients were lacking pathogenic variants in known genes implicated in IRDs, including CSNB. Two different homozygous pathogenic variants, c.1563_1566del, p.(Val522Glufs*18) and c.1795C>T, p.(Arg599*) in *EGFLAM* were identified by ES and GS. The corresponding protein is localized in the outer plexiform layer and important for ON-bipolar cell signaling in the retina.

**Conclusion and Relevance:**

This case series reports on a gene defect in *EGFLAM* implicated in human cCSNB. Clinicians should be aware about this association and consider including *EGFLAM* in diagnostic gene panels for IRDs. This discovery may lead to faster and more accurate diagnosis of cCSNB and genetic counseling, as well as a pathway for developing therapies.

## Key Points

**Question:**

What is the underlying gene defect in patients with genetically unsolved complete congenital stationary night blindness (cCSNB) representing a dysfunction of the ON-bipolar cell signaling in the retina?

**Findings:**

This case series reports on 3 patients of 2 unrelated families of Moroccan ancestry with cCSNB harboring 2 different pathogenic variants in *EGFLAM*. This gene codes for a protein localized in the outer plexiform layer, which is essential for ON-bipolar cell signaling in the retina, explaining the phenotype of these patients.

**Meaning:**

This work led to the discovery of a gene defect implicated in cCSNB to be included in the genetic analysis of inherited retinal disease cases.

## Teaser

This case series describes the phenotype and underlying gene defect in patients with complete congenital stationary night blindness from 2 unrelated families.

## Introduction

Inherited retinal disorders (IRDs) are a group of clinically and genetically heterogeneous and mainly monogenic ocular disorders with a prevalence of approximately 1 in 1380 individuals, affecting 5.5 million people worldwide.^1,2^ Today, more than 300 genes have been implicated in IRDs.^3^ However, despite advances in high-throughput sequencing and in silico prediction tools, the pathogenic variants are still unknown for approximately 30% of cases.^4^ IRDs can be progressive, associated with loss of retinal cells, or stationary, associated with a functional defect. One form of the latter is congenital stationary night blindness (CSNB). It is a clinically and genetically heterogeneous condition caused by defects in signal processing within photoreceptors, retinoid recycling in the retinal pigment epithelium or signal transmission from photoreceptors to bipolar cells (BCs) in the retina.^5^ The mode of inheritance can be autosomal dominant, autosomal recessive, or X-linked.^5^ The most distinctive clinical manifestation is impaired night or dim light vision, but other ocular symptoms can also be present, including delayed dark adaptation, photophobia, poor visual acuity, myopia, nystagmus, strabismus, and fundus abnormalities in some affected individuals.^5^ Most of the CSNB cases are of the Schubert-Bornschein subtype, characterized by electronegative electroretinogram (ERG) responses under scotopic conditions where the amplitude of the a-wave is normal but the amplitude of the b-wave is severely reduced.^5,6^ This form has been classified into 2 types, incomplete CSNB (icCSNB or CSNB2) and complete CSNB (cCSNB or CSNB1), both associated with a signal transmission impairment from photoreceptors to ON-BCs (with additional impairment of transmission to OFF-BCs in icCSNB). While under scotopic conditions in icCSNB the b-wave is only reduced, it is completely absent in cCSNB. We and others have identified pathogenic variants in *NYX*, *TRPM1*, *GRM6*, *GPR179*, and *LRIT3* leading to cCSNB.^5,7,8,9,10,11,12,13,14,15,16^ Variants in the same genes have been shown to lead to cCSNB-like phenotype in mice.^11,17,18,19,20,21,22,23,24,25,26,27,28,29,30,31,32,33,34,35^ Proteins encoded by these genes are located in the outer plexiform layer, mainly at the dendritic tips of rod and cone ON-BCs, aligned with the ON-BCs defect, resulting in the absence of the b-wave and cCSNB.

Although several cCSNB-associated genes have already been identified, there are still unsolved cases for which the genetic cause and molecular mechanism remain unknown.^4^ The purpose of our work was to report the clinical phenotype and identify the underlying gene defect in patients with genetically unsolved cCSNB using genome or exome sequencing (GS or ES). This work provides new insight into molecular mechanisms and genetic causes of cCSNB and delivers a potential target for the development of therapies.

## Methods

### Participants and Clinical Assessment

Research procedures were conducted in accordance with institutional guidelines. Study protocol adhered to the tenets of the Declaration of Helsinki and received approval from the local ethics committees for the respective families (family 1 from France and family 2 from the Netherlands). Written, informed consent was obtained from all participants prior to their inclusion in this study. No compensation or incentive was offered to patients to participate in the study. This study was reported in accordance with relevant reporting guidelines outlined by Kempen et al.^36^

Participants were identified from large historical cohorts of individuals affected by CSNB at 3 different referral centers (National Reference Centre for Rare Retinal Diseases, Quinze-Vingts Hospital, Paris; Reference Centre for Rare Ocular Diseases, Exploration de la Vision et Neuro-Ophtalmologie, Centre Hospitalier Universitaire de Lille; the Rotterdam Eye Hospital, Rotterdam). They underwent extensive ophthalmic examinations by experienced IRD specialists, including standard-of-care functional assessments including full-field ERG following the International Society for Clinical Electrophysiology of Vision standards and multimodal retinal imaging as previously described^37,38,39,40^ (detailed ERG phenotyping protocol of each center is available in Supplement 1).

### Genetic Analyses

Blood samples were obtained from patients and, when possible, their parents. Total genomic DNA was extracted from peripheral blood lymphocytes by standard procedures.

**Figure 1.**
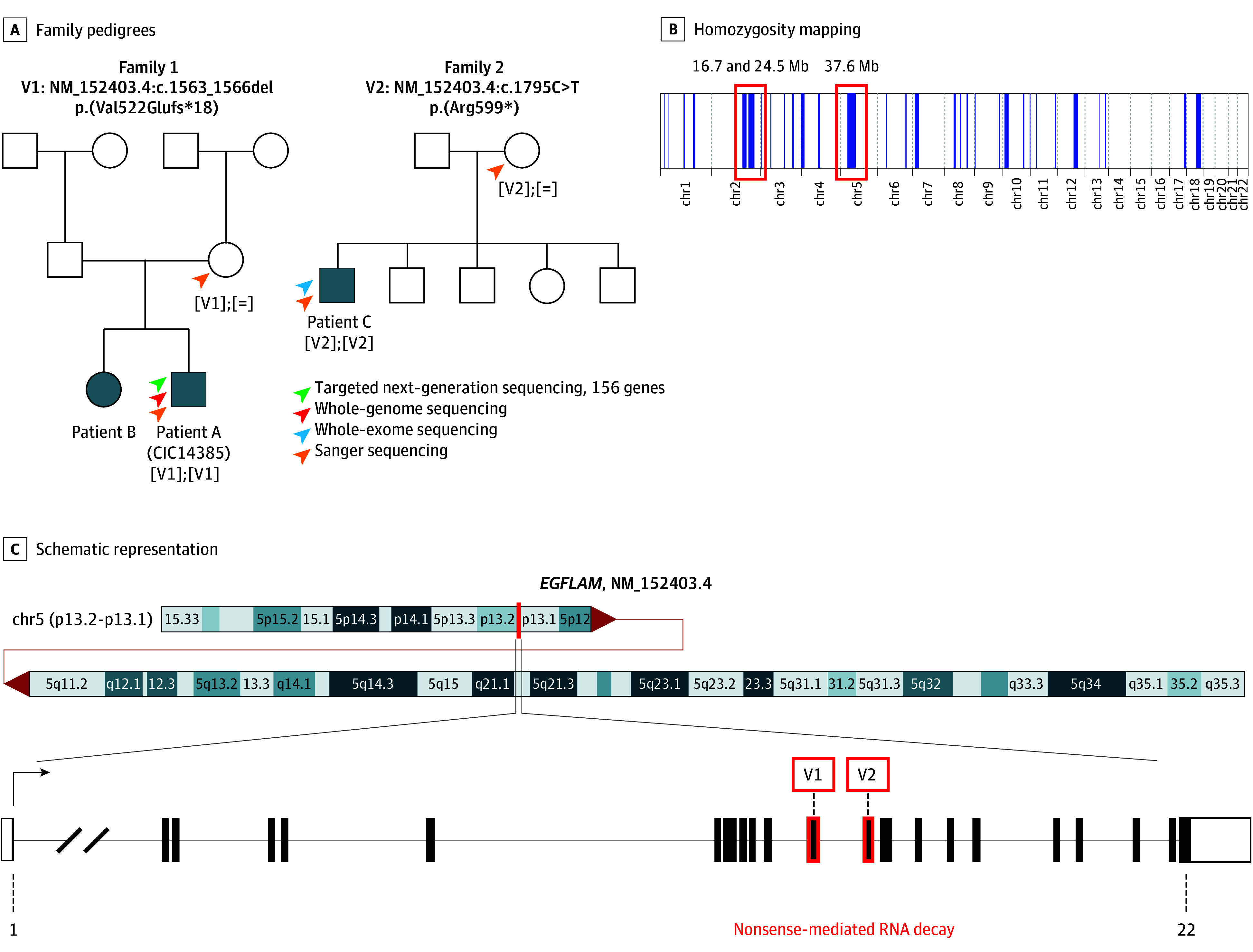
Identification of Homozygous Pathogenic Variants in *EGFLAM* in 2 Unrelated Families Affected by Autosomal-Recessive Complete Congenital Stationary Night Blindness A, Pedigrees of family 1 and family 2 analyzed in the present study. The arrowheads indicate the genetic screening performed on each individual. Homozygous deletion NM_152403.4:c.1563_1566del, p.(Val522Glufs*18) in *EGFLAM* was identified in proband A (CIC14385) and it cosegregated with the phenotype in the family. Similarly, in family 2, a homozygous NM_152403.4:c.1795C>T, p.(Arg599*) pathogenic variant was found, and it cosagregated with the phenotype. B, Homozygosity mapping using whole-genome sequencing data identified 2 large homozygous regions in proband A, found in chromosome 2 (sizes 16.7 Mb and 24.5 Mb), and chromosome 5 (size 37.6 Mb). C, Schematic representation of the structure of *EGFLAM* (RefSeq NM_152403.4; harboring 22 exons), filled and unfilled boxes represent coding and noncoding exonic regions respectively. V1 and V2 depict the positions of pathogenic variants identified in the current study.

Patient A, an affected male (CIC14385) from a consanguineous family 1 (F8170), previously excluded for known cCSNB gene defects by targeted next-generation sequencing panel of 156 IRD genes (Figure 1), underwent GS (IntegraGen) using a NovaSeq6000 with a coverage of 30×.^2^ Patient C from family 2 underwent ES (Figure 1). Data obtained in this way were bioinformatically analyzed and variants filtered down to identify the most likely pathogenic variant as previously described (eMethods in Supplement 1).^41^ Direct Sanger sequencing of putative pathogenic variants was performed on the patients and available family members for confirmation and segregation.

## Results

### Phenotype of Patients

We identified 3 patients from 2 different families which revealed typical cCSNB including high myopia. Patient A (CIC14385 from F8170) and his affected sister, patient B, from a consanguineous Moroccan family (family 1) were referred for high myopia and low visual acuity and first assessed at age 3 and 5 years, respectively. Parents reported poor visual behavior of the siblings in the darkness, and the children experienced night blindness afterwards as well. Clinical data are summarized in the Table.

**Table.** Clinical Data of Affected Individuals With Complete Congenital Stationary Night Blindness

	Individuals, sex	Age, y	BCVA, Snellen	Refraction under cycloplegia	Visual field	ffERG	Anterior segment	Fundus	SW-FAF, NIRAF, IRR	SD-OCT
Family 1 (F8170)	CIC14385 A, M	3	OU: 20/100	RE: −4.75 (−0.75) 90°; LE: −4.00 (−2.50) 120°	NA	NA	UR	Tilted discs; thinning of the retina at the posterior pole	UR	UR
		5	RE: 20/63; LE: 20/100	RE: −6.00 (−1.00) 40°; LE: −6.75 (−1.00) 130°	Kinetic: narrowed to V4e target (the only 1 tested)	DA 0.01: undetectable; DA 3 and DA 10: electronegative waveform; LA 3: square-shaped a-wave, abrupt b-wave of slightly reduced amplitude; LA 30 Hz: broadened though; ON-OFF: normal a-wave, severely reduced ON-b-wave and well-preserved OFF–d-wave	UR	Tilted discs; thinning of the retina at the posterior pole	UR	Normal layering with posterior staphyloma
	Affected sister B, F	8	OU: 20/63	RE: −8.50; LE: −9.0	NA	NA	UR	Tilted oval-shaped discs; thinning of the retina in posterior pole due to staphyloma	UR	Normal layering with posterior staphyloma
		11	RE: 20/25; LE: 20/20	RE: −10.50; LE: −11.0	Static: central hyposensitivity, MD −0.7 dB; Kinetic: normal to III4e target	DA 0.01: undetectable; DA 3 and DA 10: electronegative waveform; LA 3: square-shaped a-wave, abrupt b-wave of slightly reduced amplitude; LA 30 Hz: normal; ON-OFF: normal a-wave, severely reduced ON–b-wave and well-preserved OFF–d-wave	UR	Accentuation of retinal thinning in posterior pole	UR	NA
Family 2	C, M	13	RE: 20/25; LE 20/25	RE: −14.25 LE: −14.00	Kinetic: normal	DA 0.01 undetectable; DA 3 and DA 10: electronegative waveform; LA 3: square-shaped a-wave, abrupt b-wave, amplitudes within normal limits; LA 30 Hz: amplitude within normal limits and broadened trough; ON-OFF: NA	UR	Tilted oval-shaped discs; thinning of the retina in posterior pole due to staphyloma	NTR	Normal layering with posterior staphyloma

Abbreviations: BCVA, best-corrected visual acuity; F, female; LE, left eye; M, male; NA, not available; NTR, nothing to report; OU, both eyes; RE, right eye; UR, unremarkable.

**Figure 2.**
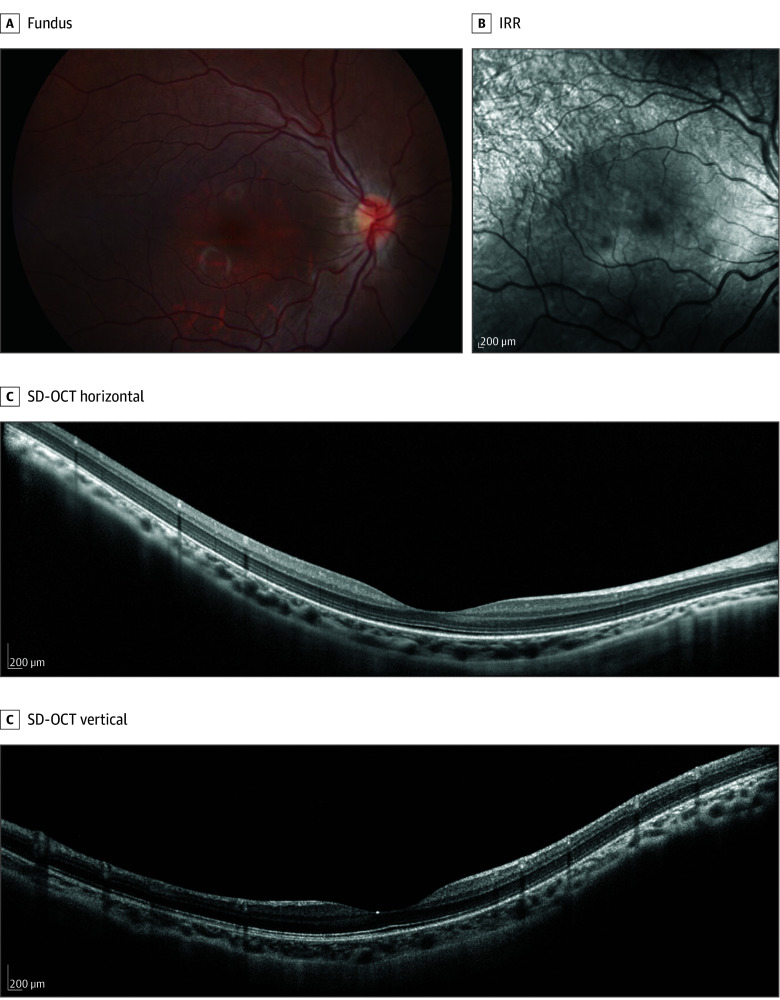
Retinal Imaging of an Individual With Autosomal-Recessive Complete Congenital Stationary Night Blindness Carrying an *EGFLAM* Pathogenic Variant Fundus examination in patient A found myopic changes, including tilted discs, posterior pole staphyloma with thin retina, and increased visibility of choroidal vasculature; there were no peripheral retinal abnormalities. Besides myopic changes, multimodal retinal imaging—infrared reflectance (IRR) and spectral-domain optical coherence tomography (SD-OCT)—results were unremarkable. Short-wavelength fundus autofluorescence and near infrared autofluorescence results are available in eFigure 1 in the Supplement.

Both patients were orthotropic without nystagmus. Anterior segment examination was unremarkable. Fundus examination found myopic changes, including tilted discs, posterior pole staphyloma with thin retina, and increased visibility of choroidal vasculature; there were no peripheral retinal abnormalities (Figure 2; eFigure 1 in Supplement 1). Multimodal retinal imaging—including short-wavelength fundus autofluorescence, near infrared autofluorescence, infrared reflectance, and spectral-domain optical coherence tomography—was unremarkable apart from myopic changes (Figure 2; eFigure 1 in Supplement 1).

**Figure 3.**
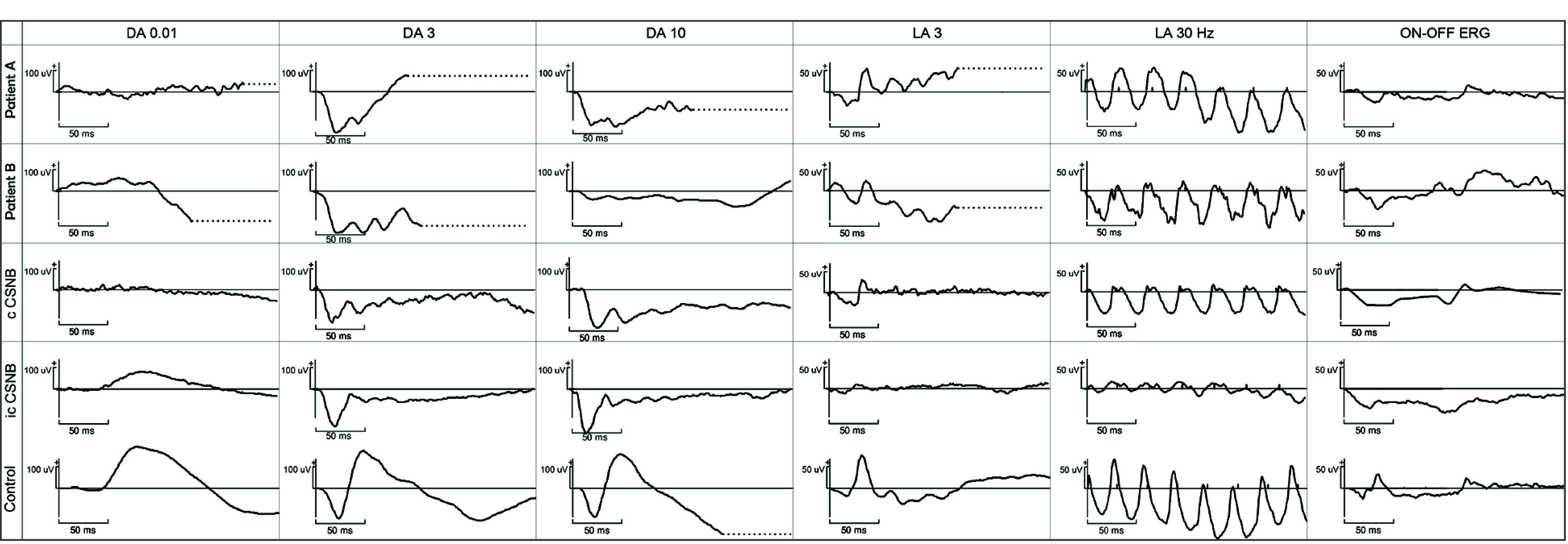
Full-Field Electroretinography Recordings (ffERG) of Individuals With Autosomal-Recessive Complete Congenital Stationary Night Blindness (cCSNB) in Family 1 Carrying an *EGFLAM* Pathogenic Variant In patients A and B, there were no responses to DA 0.01, and flashes of increasing intensity (DA 3 and DA 10 ERG) showed a normal a-wave but a severely reduced b-wave, giving an electronegative waveform configuration. Light-adapted response to 3 cd·s/m^2^ flash (LA 3) showed a distinct square-shaped a-wave, with a sharply rising b-wave of slightly reduced amplitude; light-adapted flicker (LA 30 Hz) was normal in amplitude but delayed in period. Responses to long-duration flashes (ON-OFF ERG) showed a normal a-wave and severely reduced ON-b-wave with a well-preserved OFF–d-wave. For comparison, ffERG of an individual with cCSNB harboring an *NYX* gene defect (3rd row) and incomplete CSNB (icCSNB) of an individual harboring a *CACNA1F* gene defect (4th row) are shown. Overall ffERG traces of patients were in keeping with a Schubert-Bornschein complete type of CSNB.

Full-field ERG (ffERG) recordings under dark-adapted conditions showed no responses to a dim 0.01 cd·s/m^2^ flash (DA 0.01) and a normal a-wave but a severely reduced b-wave, giving an electronegative waveform configuration to bright flashes (DA 3 and DA 10). Light-adapted ffERG to 3 cd·s/m^2^ flash (LA 3) showed a peculiar square-shaped a-wave configuration with sharply rising b-wave of slightly reduced amplitude; light-adapted flicker (LA 30 Hz) was normal in amplitude with a delayed period and a broadened trough. Responses to long-duration flashes (ON-OFF ERG) showed a normal a-wave but a severely reduced ON–b-wave and a well-preserved OFF–d-wave. Overall ffERG was in keeping with Schubert-Bornschein complete type (Figure 3).

In addition, patient A had severe congenital hearing loss due to a homozygous *GJB2* pathogenic variant (NM_4004.6c.35del, p[Gly12Valfs*2]) and underwent cochlear implantation.^42^ The siblings were last assessed at the age of 8 and 11 years, respectively (Table). Night blindness complaints were still present. Their myopia had progressed. Best-corrected visual acuity was still low for patient A (CIC14385, F8170) but had become near normal in the affected sibling B (right eye: 20/25 and left eye: 20/20). Slitlamp and fundus examination results had not changed since their first visit. Both children were following a normal education at school.

Patient C from family 2 was referred at age 4 years for high myopia and low visual acuity (Table). He had orthotropia, there was no nystagmus, and at first there were no instances of night blindness. The anterior segment was unremarkable, and the fundus displayed tilted or myopic discs and peripapillary chorioretinal atrophy (eFigure 1 in Supplement 1). At age 13 years, his ffERG resembled those of patient A and B: a Schubert-Bornschein cCSNB type with an electronegative waveform to dark-adapted bright flash DA 3 and the squared a-wave and abrupt b-wave on the light-adapted LA 3 (eFigure 2 in Supplement 1). By this time, the individual was experiencing night blindness. At his last clinical examination at age 22 years, he had a subnormal visual acuity of 20/32. The anterior segment was still normal. Funduscopy displayed mild myopic staphyloma with increased visibility of choroidal vasculature due to thinning of the retinal pigment epithelium. The retinal periphery was unremarkable. His kinetic visual field was normal (eFigure 3 in Supplement 1). For patient C, no consanguineous relationship between the parents was reported; however, parents were from the same town in Morocco.

### Identification of 2 Different Rare *EGFLAM* Pathogenic Variants Associated With cCSNB

In patient A (CIC14385 in F8170), in whom pathogenic variants in known CSNB genes were excluded previously, GS identified a homozygous deletion of 4 bases in exon 12 of *EGFLAM* NM_152403.4:c.1563_1566del, p.(Val522Glufs*18) (Figure 1A). No other likely pathogenic variant was found, as all the other homozygous variants were either too frequent or predicted to be tolerated. The identified pathogenic variant in *EGFLAM* was predicted to be deleterious by the in silico prediction program CADD (35) and which was absent from gnomAD (v4.1.0) allele frequency database (eTable in Supplement 1). The pathogenic variant was located in a large region of homozygosity on chromosome 5 (37.6 Mb; Figure 1B), containing *EGFLAM* (Figure 1C) and cosegregated with the phenotype in all available family members (Figure 1A).

ES identified another homozygous nonsense pathogenic variant in exon 13 of *EGFLAM* in patient C, also diagnosed with cCSNB, NM_152403.4:c.1795C>T, p.(Arg599*), and which also cosegregated with the phenotype in all available family members (Figure 1A). It was predicted to be deleterious by CADD (44.0) and BayesDel (0.45) and was absent from gnomADv2 but showed a very low allele frequency of 3.10E-06 in gnomADv4 (eTable in Supplement 1). Bioinformatic analysis predicted that both pathogenic variants may trigger nonsense-mediated mRNA decay.

## Discussion

Presently, there are more than 350 reported pathogenic variants in 5 genes (*NYX*, *TRPM1*, *GRM6*,* GPR179*, and *LRIT3*) leading to cCSNB.^5,43^ The global prevalence database counting only the different type of pathogenic variants or affected alleles show 155 pathogenic variants in *NYX*, 112 pathogenic variants in *TRPM1*, 68 pathogenic variants in *GRM6*, 18 pathogenic variants in *GPR179* and 7 pathogenic variants in *LRIT3* (Human Gene Mutation Database Professional 2025.1 database).^44^ These genes code for proteins located in the outer plexiform layer of the retina.^5^ However, there are still several cases lacking pathogenic variants in known gene defects. Even though technological advances theoretically allow for rapid gene defect identification, it remains challenging due to variants that are often rare and may only be observed within a single family.^4,45^

The 3 patients of Moroccan origin in this study presented with typical cCSNB Schubert-Bornschein type of ERGs representing a complete ON-bipolar cell dysfunction. Other ocular signs found in cCSNB including high myopia were present except nystagmus.

In this case series, we identified 2 different homozygous truncating pathogenic variants in *EGFLAM* in those patients, which were predicted to lead to nonsense-mediated mRNA decay and thus to the absence of *EGFLAM*. It was ultimately identified as a very rare genetic defect, which may be explained by the overall rarity of CSNB—partly due to the specific clinical protocols required for accurate diagnosis. Alternatively, this gene defect may be more prevalent in individuals of Moroccan descent or more broadly of African descent, where clinical and genetic diagnostics are less frequently performed. Further genetic studies involving previously unexamined cohorts could provide valuable insights into this finding.

**Figure 4.**
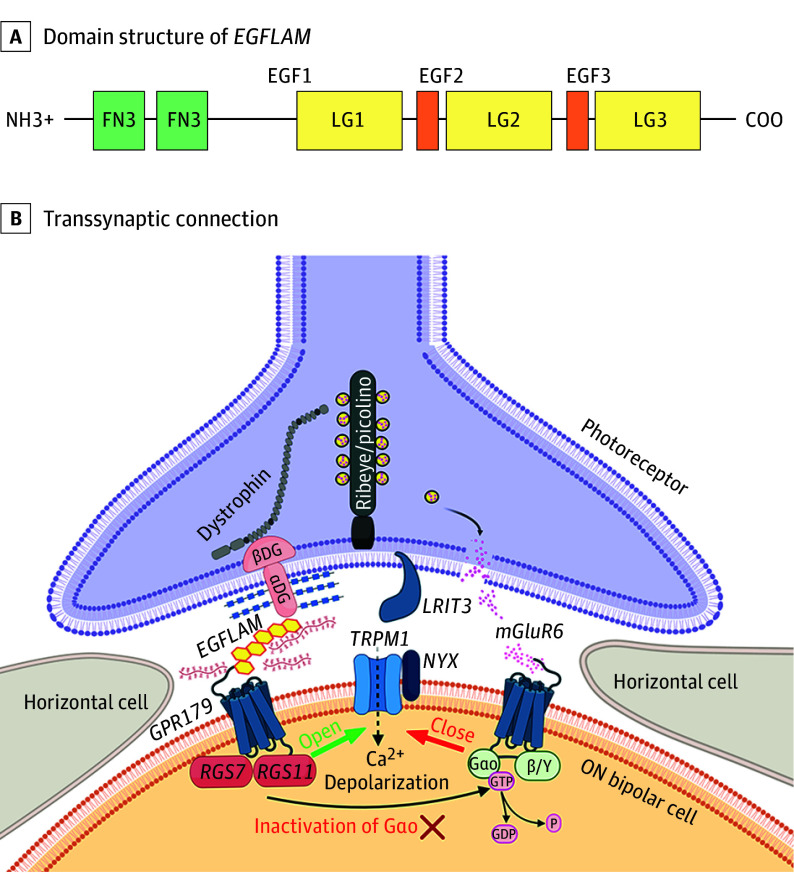
Role of *EGFLAM* in Photoreceptor Ribbon Synapse Organization and Domain Structure A, Representation of the domain structure of *EGFLAM*. On the N-terminus, there are 2 fibronectin type III domains (FN3), and on the C-terminus, 3 EGF-like and 3 laminin G-like domains (EGF-LG). B, *EGFLAM* forms a transsynaptic connection by interacting with the dystroglycan-dystrophin complex on the photoreceptor side and GPR179 located on the ON-bipolar cell membrane. Molecules already known to be implicated in complete congenital stationary night blindness are represented in blue (NYX, TRPM1, GRM6, GPR179, and LRIT3).

The *EGFLAM* (NM_152403.4) gene is located on chromosome 5p13.2-p13.1 and consists of 22 coding exons (Figure 1C), coding for EGF-like, fibronectin type III, and laminin G domains protein (EGFLAM), also known as Pikachurin. The protein contains 1009 amino acids, which form 2 fibronectin type III domains at the N-terminus and 3 EGF-like/laminin G-like domains at the C-terminus (Figure 4A). EGFLAM represents an extracellular matrix protein, which enables precise interaction between photoreceptors and BC dendrites through its interactions with the dystroglycan-dystrophin complex located in photoreceptor cells, and GPR179 localized in ON-BC (Figure 4B) whose variants have already been implicated in cCSNB.^7,11,46^ Similar to other heparin sulfate proteoglycans, EGFLAM interacts with α-dystroglycan via laminin G-like (LG) domains LG2 and LG3 which together form specific steric structure enabling this interaction.^47,48,49,50^ Via the third LG domain, EGFLAM interacts with the cache domains of GPR179 by forming a tetrameric assembly.^46^ These extensive and costabilizing interactions seem to be initiated by EGFLAM, which localizes at the presynaptic site preceding GPR179 postsynaptic recruitment.^31^ Indeed, studies from mice lacking EGFLAM showed that it is important for the correct localization in the dendritic tips of postsynaptic proteins including RGS7, RGS11, and GPR179.^31^ Notably, *Egflam *knockout mice show a phenotype similar to the one seen in mouse and human cCSNB.^5,49^

Transcriptomic databases revealed high tissue-specific *EGFLAM* expression in the retina (Human Protein Atlas; eFigure 4A in Supplement 1) and more specifically in both rod and cone photoreceptors.^51^ Additionally, immunohistochemistry localized EGFLAM in the outer plexiform layer (Human Protein Atlas; eFigure 4B in Supplement 1).^51^ Photoreceptor cell expression of *Egflam* was confirmed in the mouse retinal-cell-type comparative transcriptome atlas (eFigure 4C in Supplement 1) and via the in-house rd1 mouse transcriptomic database (rd1 mouse line, Internal database of Institut de la Vision; eFigure 4D in Supplement 1).^52,53^ Single-cell RNA sequencing database suggested *Egflam* expression mainly in cone photoreceptors but also in several BCs (eFigure 4E in Supplement 1).^54^ In summary, database analysis revealed that *EGFLAM* is mainly expressed in the photoreceptors and possibly also in BCs. These findings were unexpected, since most of the other genes implicated in cCSNB are solely expressed in ON-BCs.^5,55^ The expression profile of *EGFLAM* corresponds more to other genes implicated in icCSNB, such as *CACNA1F*, expressed in the synapses of rods and cones, leading to a dysfunction of the ON-and OFF-pathway, which can be explained by the fact that cone photoreceptors interact with both ON- and OFF-BC.^56,57^ However, in the individuals with CSNB and *EGFLAM* pathogenic variants in this study, only the ON pathway was impacted. The OFF pathway remained intact, showing ffERG with conserved photopic responses and normal OFF responses in long duration stimulations, unlike what is found in icCSNB.^58^ Similarly, an individual in our study showed a preserved i-wave, which was also thought to originate from the OFF-pathway.^59^ So, although *EGFLAM* is expressed in cones, patients with pathogenic variants in *EGFLAM* may reveal cCSNB due to EGFLAM’s interaction with GPR179, also implicated with cCSNB and located specifically in the dendritic tips of ON-BCs.^7,11^

In addition to the specific ERG phenotype found in individuals with ON-BC defect, high myopia, commonly present in individuals with cCSNB, was also detected. It is known that signaling involving positive glutamatergic input coming from ON-BCs to dopaminergic amacrine cells represents the primary driver of dopaminergic signaling in the inner retina.^60,61,62,63,64^ Similarly, animal models and human epidemiological studies have shown that dopamine acts as a negative regulator of eye growth during emmetropization, and lack of it seems to be correlated with myopia.^58,61,65^ Considering this, we can hypothesize that aberrantly folded or absent EGFLAM, leading to dysfunctional photoreceptor synapse and absence of ON-BC input to dopaminergic amacrine cells, could be the reason of the appearance of high myopia in cCSNB.

Previously, we showed that cCSNB pathogenic variants p.(Tyr220Cys), p.(Gly455Asp), and p.(His603Tyr) in *GPR179* caused trafficking defects, resulting in cell surface mislocalization and ON-BC signaling defect.^24^ This was not the case for another cCSNB-causing pathogenic variant, p.(Asp126His), affecting the N-terminal part of the protein, and we hypothesized that the p.(Asp126His) pathogenic variant is associated with the loss of a GPR179 ligand binding capacity, which was yet to be identified.^24^ Pathogenic variant p.(Asp126His) localizes in the EGFLAM-interacting part of GPR179, the α2 helix, which also represents part of GPR179 extracellular domain dimeric interface.^46^ It has been shown that EGFLAM does not act as a GPR179 ligand, and its binding does not influence kinetics of GPR179-RGS7 activity. However, it still represents an auxiliary subunit of GPR179 essential for optimal synaptic insertion, adequate conformational structure, and positioning of intracellular RGS complex.^46,66^ Binding of EGFLAM LG3 domains to cache domains of GPR179 likely occurs in the context of dimerization.^46^ As mentioned, p.(Asp126His) variant affects extracellular domain domains of GPR179 which are important for dimerization.^46^ Therefore, we can predict that the p.(Asp126His) variant affecting extracellular domain domains of GPR179 disrupts its binding to EGFLAM and dimerization promoted through this interaction, leading to the aberration of GPR179 function and loss of RGS complexes recruitment and dysfunction of ON-BCs.

### Limitations

The primary limitation of this study is that it identified *EGFLAM* pathogenic variants in only 2 different families. This makes it difficult to determine the frequency or clinical relevance of this genetic defect.

## Conclusions

To summarize, in this case series, we identified biallelic *EGFLAM* pathogenic variants potentially leading to autosomal recessive cCSNB in 2 families. Previous work deciphering the structure of GPR179 and its complex interactions with EGFLAM yielded a better understanding of the molecular mechanism underlying the observed cCSNB phenotype. With this in mind, we hope that our findings, which provide insight into previously unknown potential genetic causes of human cCSNB, will be a starting point for developing therapeutic approaches that address the visual impairment in affected persons.

## Notes

Supplement 1. eMethods eTable. Annotation and pathogenic predictions of EGFLAM variants found in association with congenital stationary night blindness eFigure 1. Retinal imaging of autosomal-recessive cCSNB individuals carrying EGFLAM pathogenic variants eFigure 2. Full-field electroretinography (ffERG) of autosomal-recessive cCSNB individual carrying a EGFLAM mutation (Patient C) eFigure 3. Kinetic visual fields of Patient C eFigure 4. Analysis of EGFLAM mRNA expression and protein localization in retina, confirming the localization in the outer plexiform layer (OPL) eReferences

Supplement 2. Data sharing statement
