# Supplementary material for: EGFLAM Pathogenic Variants and Congenital Stationary Night Blindness
Source: JAMA Ophthalmol. 2025 Dec 4;144(1):79–88. doi: 10.1001/jamaophthalmol.2025.4888 (PMC12679428; doi:10.1001/jamaophthalmol.2025.4888)
Supplement: Supplement 1. — eMethods eTable. Annotation and pathogenic predictions of EGFLAM variants found in association with congenital stationary night blindness eFigure 1. Retinal imaging of autosomal-recessive cCSNB individuals carrying EGFLAM pathogenic variants eFigure 2. Full-field electroretinography (ffERG) of autosomal-recessive cCSNB individual carrying a EGFLAM mutation (Patient C) eFigure 3. Kinetic visual fields of Patient C eFigure 4. Analysis of EGFLAM mRNA expression and protein localization in retina, confirming the localization in the outer plexiform layer (OPL) eReferences [file jamaophthalmol-e254888-s001.pdf]

Supplemental Online Content

Boranijasevic S, Smirnov V, Navarro J, et al. *EGFLAM* pathogenic variant and congenital stationary night blindness. *JAMA Ophthalmol*. Published online December 4, 2025. doi:10.1001/jamaophthalmol.2025.4888

eMethods

eTable. Annotation and pathogenic predictions of EGFLAM variants found in association with congenital stationary night blindness

eFigure 1. Retinal imaging of autosomal-recessive cCSNB individuals carrying EGFLAM pathogenic variants

eFigure 2. Full-field electroretinography (ffERG) of autosomal-recessive cCSNB individual carrying a EGFLAM mutation (Patient C)

eFigure 3. Kinetic visual fields of Patient C

eFigure 4. Analysis of EGFLAM mRNA expression and protein localization in retina, confirming the localization in the outer plexiform layer (OPL)

eReferences

This supplemental material has been provided by the authors to give readers additional information about their work.

© 2025 Boranijasevic S et al. *JAMA Ophthalmology*.

Supplemental material

eMethods

ERG phenotyping procedure

Electroretinograms (ERGs) of Patient A and Patient B were obtained with Metrovision MonColor unit (Metrovision, Perenchies, France) in accordance with the standards of the International Society for Clinical Electrophysiology of Vision (ISCEV) adapted to pediatric purpose. We used adhesive skin electrodes; recording bandwidths of 1–49 Hz for all stimulations but light-adapted 30 Hz flicker (1-300 Hz); 50Hz band was rejected.

For Patient C ERGs were obtained in accordance with the standards of ISCEV, with recording bandwidths of 0.3–300 Hz. Signals were digitized at a sampling frequency of 2 kHz per channel. Dawson–Trick–Litzkow (DTL) electrodes served as corneal electrodes and were positioned just inferior to the cornea along the lower eyelid margin. Flash stimuli were delivered using an Espion E system equipped with a ColorDome stimulator (Diagnosys LLC, Cambridge, United Kingdom). Recordings were performed with Diagnosys Espion software (version 6.2015.428.54).

Bioinformatic analysis

Burrows-Wheeler Aligner (BWA) tool was used to map the reads to the Human genome build (hg38). SNVs and small indels calling was performed via Broad Institute's GATK Haplotype Caller GVCF tool (GATK 4.1.7.0) and the Ensembl's VEP (Variant Effect Predictor, release VEP 95.1) program processed the variants for further annotation. The regions of homozygosity (RoHs) were established by performing homozygosity mapping with AutoMap (1.3) using the SNVs.[1] Variants were prioritized on the basis of a minor

© 2025 Boranijasevic S et al. *JAMA Ophthalmology*.

allele frequency (MAF) ≤ 0.0005 in the genome Aggregation Database (https://gnomad.broadinstitute.org/ gnomAD v4.1.0), representing insertions or deletions (InDels), nonsense, missense, splice site variants and assessed for their pathogenicity through bioinformatic tools including SpliceAI, REVEL, CADD v1.7, BayesDel the score ranges from -1.29334 to 0.75731, with higher scores corresponding to a greater risk of pathogenicity and MetaRNN deleteriousness meta-scores, which integrate multiple *in silico* predictors, allele frequency information, and conservation scores (only for MetaRNN).[2-6] In the pipeline, we also introduced AlphaMissense, an artificial intelligence-based approach to predict the pathogenicity of missense variants based on the protein structure prediction tool AlphaFold.[7,8]

In the Patient C from the Family 2 exome sequencing (ES) was performed using an Illumina NovaSeq 6000, after enrichment of the exome with the Twist Exome 2.0 plus Comprehensive Exome Spike-in Kit. Read alignment was carried out with BWA, and variant calling was performed using GATK for single nucleotide variants (SNVs) and CoNIFER and/or ExomeDepth for copy number variants (CNVs). Subsequently, the variants were annotated by the Genetics departments of Radboudumc and MaastrichtUMC+ using an in-house developed pipeline. Via an in-house made tool exomes of 2730 individuals with visual impairment were analysed at the Radboudumc, and were screened for variants in *EGFLAM*, showing a frequency of <1% in gnomAD v4.1.0. The Patient C of the Family 2 was the only individual detected with biallelic variants in *EGFLAM*. Sanger sequencing was performed on DNA of the parents for segregation analysis.

© 2025 Boranijasevic S et al. *JAMA Ophthalmology*.

Expression analysis

The expression of the *EGFLAM* gene was investigated using publicly and in-house available databases (https://www.proteinatlas.org/ENSG00000164318-EGFLAM, https://www.fmi.ch/roska.data/index.php, http://kbass.institut-vision.org/KBaSS/transcriptomics/GraphManager.php, https://singlecell.broadinstitute.org/single_cell/study/SCP3/retinal-bipolar-neuron-drop-seq?genes=Egflam&tab=distribution#study-visualize) as previously described.[9]

© 2025 Boranijasevic S et al. *JAMA Ophthalmology*.

eTables and eFigures

eTable. Annotation and pathogenic predictions of *EGFLAM* variants found in association with congenital stationary night blindness.

**Variant description**	Genomic coordinates (NC_000005.10)	g.38418134_38418137del	g.38425077C>T
	Nucleotide change (NM_152403.4)	c.1563_1566del	c.1795C>T
	Protein change (NP_689616.2)	p.(Val522Glufs*18)	p.(Arg599*)
	Exon	Exon 12	Exon 13
	Coding impact	Frameshift	Nonsense
**Population database (gnomAD v4.1)**	Allele frequency	Not found	3.10E-06
	Homozygous Allele count	-	
	Heterozygous Allele count	-	5
***In silico* predictions**	CADD	35	44
	BayesDel	-	0.45
**ACMG**	Classification	Pathogenic	Pathogenic
	Criteria	PS3, PM2, PM3, PP1, PP3	PS3, PM2, PM3, PP1, PP3

ACMG, American College of Medical Genetics

PP1-segregation, PP3-deleterious, PP4-single genetic etiology, PP5-reputable sources, PM1-variant in hotspot, PM2-low AF, PM3-HOM or HTZ comp for recessive, PM6-De novo, PS3-functional studies, PS4-for dominant, PVS1-null variant (stop or frameshift), is a known mechanism of disease

© 2025 Boranijasevic S et al. *JAMA Ophthalmology*.

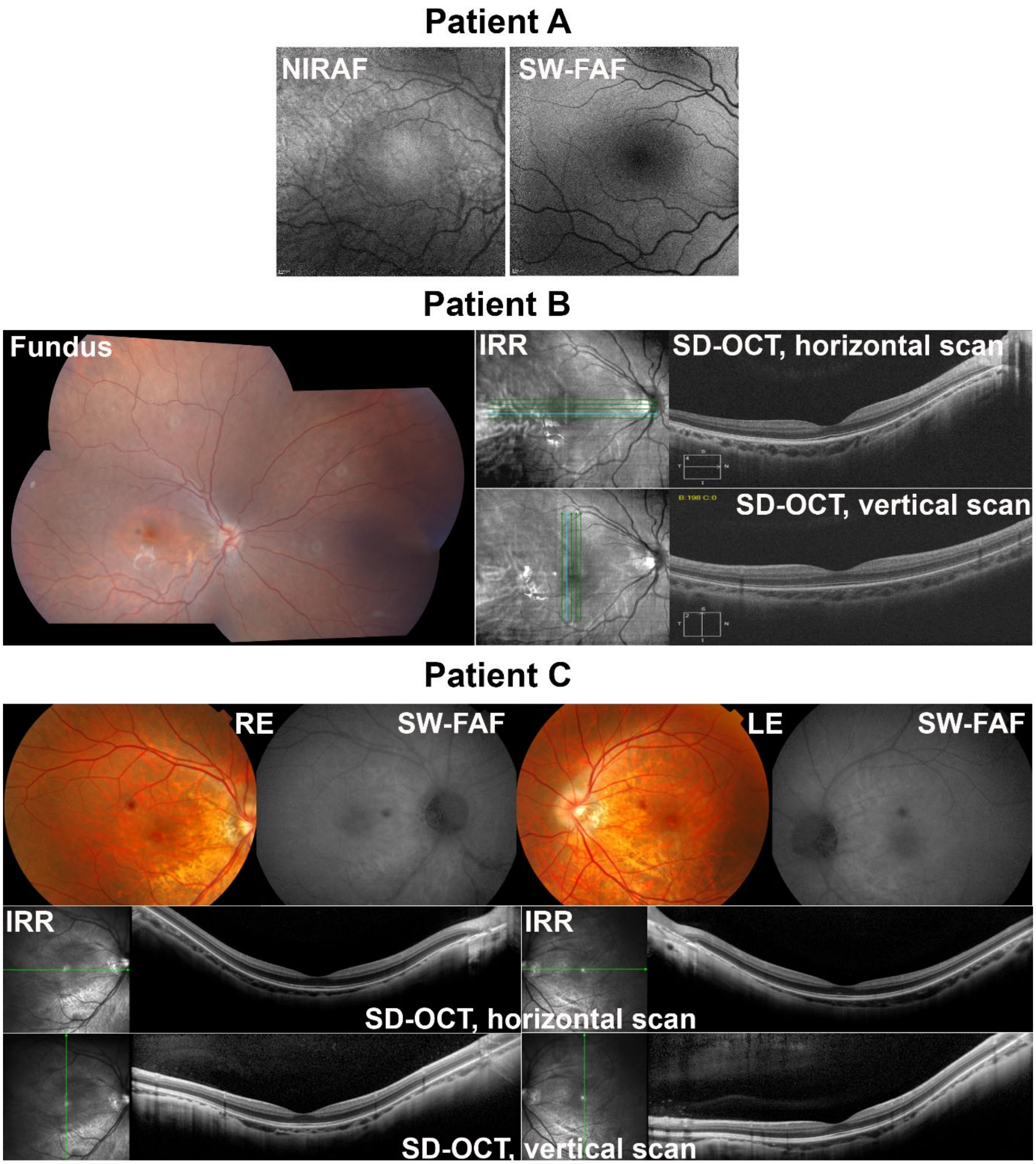

eFigure 1 Retinal imaging of autosomal-recessive cCSNB individuals carrying *EGFLAM* pathogenic variants.

© 2025 Boranijasevic S et al. *JAMA Ophthalmology*.

Retinal imaging of Patients A (more retinal imaging to be found in the main text) and Patient B from Family 1, and Patient C from unrelated Family 2. Fundus examination found myopic changes: tilted discs, posterior pole staphyloma with thin retina and increased visibility of choroidal vasculature; there were no peripheral retinal abnormalities. Besides myopic changes, multimodal retinal imaging including SW-FAF, NIRAF, IRR and SD-OCT was unremarkable.

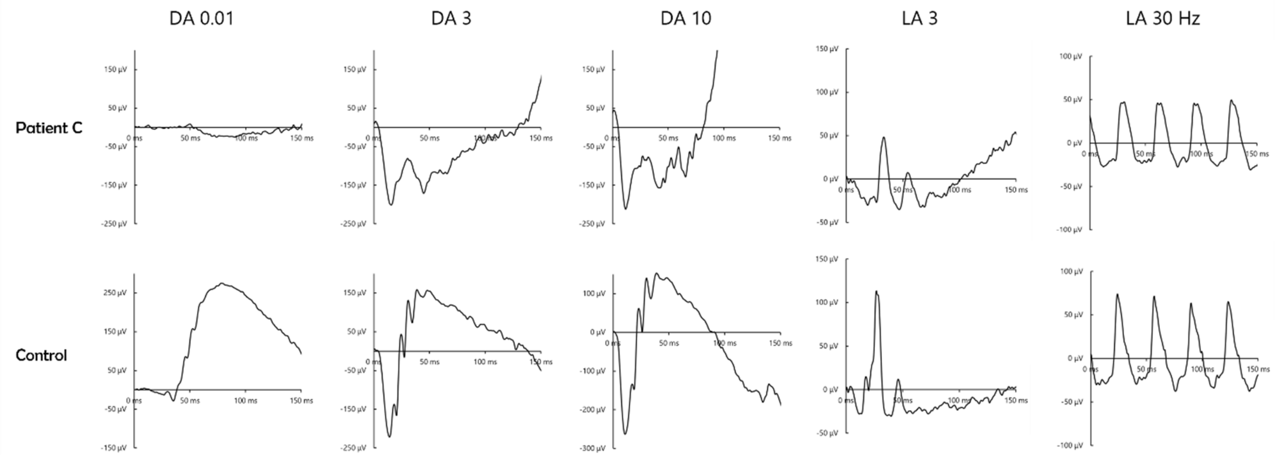

eFigure 2 Full-field electroretinography (ffERG) of autosomal-recessive cCSNB individual carrying a *EGFLAM* mutation (Patient C).

Undetectable responses to DA 0.01, electronegative waveform at the DA 3 and 10, square-shape a-wave at the LA 3, Normal amplitudes but delayed LA 30 Hz flicker responses with a broadened trough.

© 2025 Boranijasevic S et al. *JAMA Ophthalmology*.

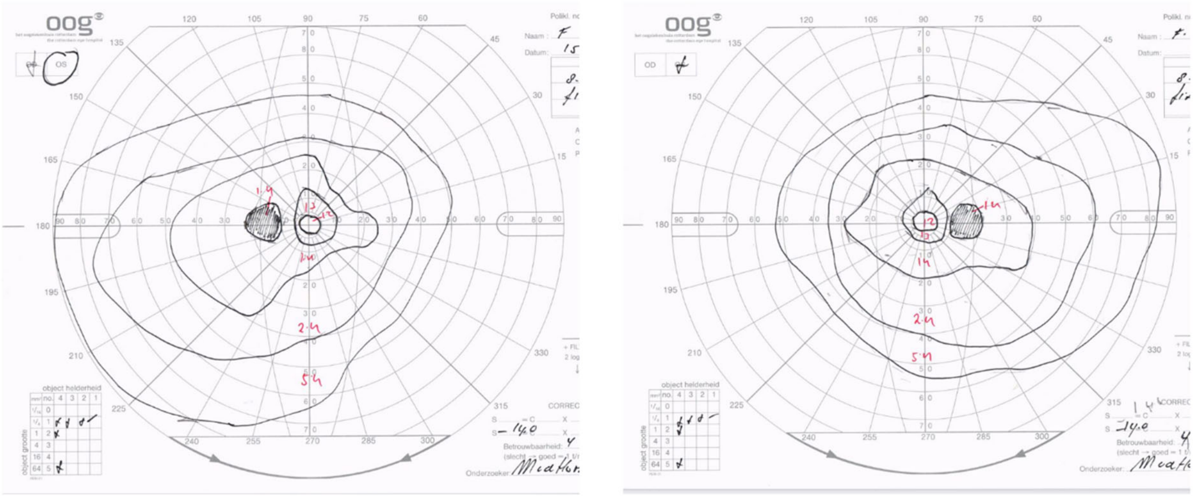

eFigure 3 **Kinetic visual fields of Patient C**

© 2025 Boranijasevic S et al. *JAMA Ophthalmology*.

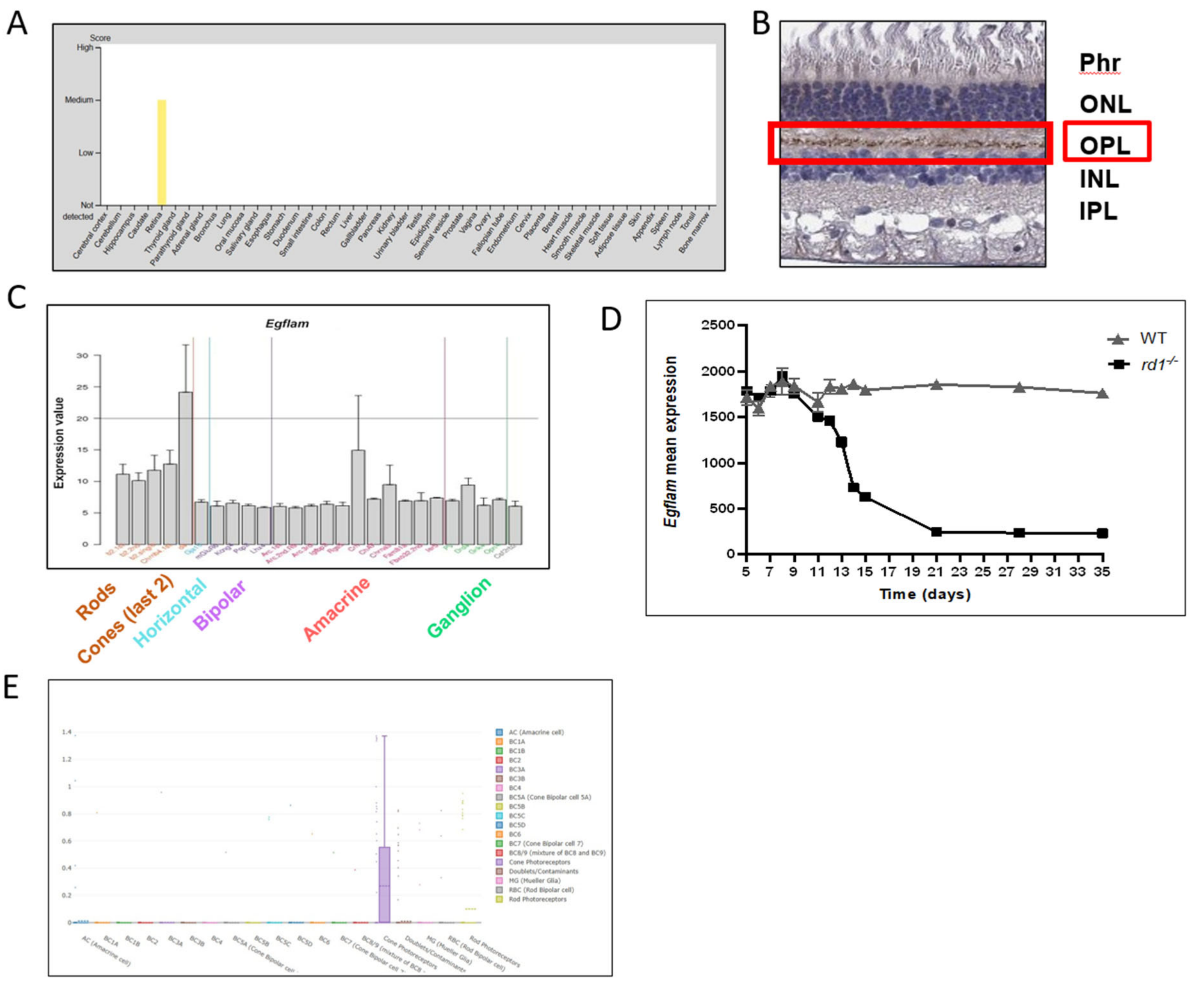

eFigure 4 Analysis of *EGFLAM* mRNA expression and protein localization in retina, confirming the localization in the outer plexiform layer (OPL).

(A) Protein expression database shows high tissue specific expression in the retina. (B) Immunohistochemical staining in human retina shows strong positivity in OPL. (C) *Egflam* expression in six different cell types from the mouse adult retina: rod photoreceptors, cone photoreceptors, horizontal cells, bipolar cells, amacrine cells, ganglion cells, and microglia cells. The graph presents *Egflam* normalized expression values. Only values higher than 20 can be considered significantly expressed. Retinal cell types were established from a library composed of 22 transgenic mouse lines. (D)

© 2025 Boranijasevic S et al. *JAMA Ophthalmology*.

Egflam expression in *rd1* and wild-type mice during photoreceptor degeneration. The *rd1* mouse, carrying *Pde6b* mutations, is a naturally occurring rod-cone dystrophy model leading to a complete loss of rod photoreceptors by postnatal day 36 and a preserved inner retina. cDNAs of neural retinas from *rd1* and wild-type mice on identical genetic backgrounds were hybridized to the mouse genome 430 2.0 array (Affymetrix). *$p < 0.05$. (E) Single-cell RNA seq database shows high expression in cone photoreceptors.

© 2025 Boranijasevic S et al. *JAMA Ophthalmology*.

eReferences:

1. Quinodoz M, Peter VG, Bedoni N, et al. AutoMap is a high performance homozygosity mapping tool using next-generation sequencing data. *Nat Commun*. Jan 22 2021;12(1):518. doi:10.1038/s41467-020-20584-4
2. Jaganathan K, Kyriazopoulou Panagiotopoulou S, McRae JF, et al. Predicting Splicing from Primary Sequence with Deep Learning. *Cell*. Jan 24 2019;176(3):535-548 e24. doi:10.1016/j.cell.2018.12.015
3. Ioannidis NM, Rothstein JH, Pejaver V, et al. REVEL: An Ensemble Method for Predicting the Pathogenicity of Rare Missense Variants. *Am J Hum Genet*. Oct 6 2016;99(4):877-885. doi:10.1016/j.ajhg.2016.08.016
4. Schubach M, Maass T, Nazaretyan L, Roner S, Kircher M. CADD v1.7: using protein language models, regulatory CNNs and other nucleotide-level scores to improve genome-wide variant predictions. *Nucleic Acids Res*. Jan 5 2024;52(D1):D1143-D1154. doi:10.1093/nar/gkad989
5. Tian Y, Pesaran T, Chamberlin A, et al. REVEL and BayesDel outperform other in silico meta-predictors for clinical variant classification. *Sci Rep*. Sep 4 2019;9(1):12752. doi:10.1038/s41598-019-49224-8
6. Li C, Zhi D, Wang K, Liu X. MetaRNN: differentiating rare pathogenic and rare benign missense SNVs and InDels using deep learning. *Genome Med*. Oct 8 2022;14(1):115. doi:10.1186/s13073-022-01120-z
7. Cheng J, Novati G, Pan J, et al. Accurate proteome-wide missense variant effect prediction with AlphaMissense. *Science*. Sep 22 2023;381(6664):eadg7492. doi:10.1126/science.adg7492
8. Jumper J, Evans R, Pritzel A, et al. Highly accurate protein structure prediction with AlphaFold. *Nature*. Aug 2021;596(7873):583-589. doi:10.1038/s41586-021-03819-2
9. El Shamieh S, Neuille M, Terray A, et al. Whole-exome sequencing identifies KIZ as a ciliary gene associated with autosomal-recessive rod-cone dystrophy. *Am J Hum Genet*. Apr 3 2014;94(4):625-33. doi:10.1016/j.ajhg.2014.03.005

© 2025 Boranijasevic S et al. *JAMA Ophthalmology*.
