# Supplementary material for: EGFLAM Pathogenic Variants and Congenital Stationary Night Blindness
Source: JAMA Ophthalmol. 2025 Dec 4;144(1):79–88. doi: 10.1001/jamaophthalmol.2025.4888 (PMC12679428; doi:10.1001/jamaophthalmol.2025.4888)
Supplement: Supplement 2. — Data sharing statement [file jamaophthalmol-e254888-s002.pdf]

Data Sharing Statement

Boranijasevic. EGFLAM Pathogenic Variants and Congenital Stationary Night Blindness. *JAMA Ophthalmol*. Published December 04, 2025. doi:10.1001/jamaophthalmol.2025.4888

Data
Data available: Yes
Data types: Deidentified participant data, Data (not involving human participants)
How to access data: christina.zeitz@inserm.fr, Institut de la Vision - Sorbonne Universités 17, Rue Moreau, 75012, Paris, France
When available: With publication

Supporting Documents
Document types: Informed consent form
How to access documents: christina.zeitz@inserm.fr, Institut de la Vision - Sorbonne Universités 17, Rue Moreau, 75012, Paris, France
When available: With publication

Additional Information
Who can access the data: Anyone
Types of analyses: For a specified purpose
Mechanisms of data availability: After approval of a proposal
